# The Added Value of Water, Sanitation, and Hygiene Interventions to Mass Drug Administration for Reducing the Prevalence of Trachoma: A Systematic Review Examining

**DOI:** 10.1155/2013/682093

**Published:** 2013-08-07

**Authors:** Anyess Travers, Sheryl Strasser, Stephanie L. Palmer, Christine Stauber

**Affiliations:** ^1^Institute of Public Health, Georgia State University, P.O. Box 3995, Atlanta, GA 30302-3995, USA; ^2^Rollins School of Public Health, Emory University, 1518 Clifton Rd NE, Atlanta, GA 30322, USA

## Abstract

Trachoma is the leading cause of infectious blindness worldwide. The SAFE strategy, the World Health Organization-recommended method to eliminate blinding trachoma, combines developments in water, sanitation, surgery, and antibiotic treatment. Current literature does not focus on the comprehensive effect these components have on one another. The present systematic review analyzes the added benefit of water, sanitation, and hygiene education interventions to preventive mass drug administration of azithromycin for trachoma. Trials were identified from the PubMed database using a series of search terms. Three studies met the complete criteria for inclusion. Though all studies found a significant change in reduction of active trachoma prevalence, the research is still too limited to suggest the impact of the “F” and “E” components on trachoma prevalence and ultimately its effects on blindness.

## 1. Introduction

Caused by the bacteria *Chlamydia trachomatis*, trachoma is the world's leading cause of infectious blindness [1]. Associated with a lack of access to water, sanitation, and hygiene education (WASH), trachoma debilitates some of the world's most marginalized peoples [2]. The World Health Organization (WHO) estimates that 325 million people in 53 countries live in trachoma-endemic areas [3]. The routes of transmission of trachoma, all of which are hygiene related, include indirect infection by fly vector as well as direct person-to-person and indirect contacts via fomites including clothing, towels, and handkerchiefs [3]. Blindness due to trachoma is caused by repeated infection of *Chlamydia trachomatis* resulting in the inflammation of the upper eyelid, which eventually leads to scarring. Scarring, over time, constricts the upper lid causing the eye lashes to pull inward, scratching, and tearing the cornea resulting in loss of vision [4, 5]. Trachoma has caused visual impairment in 2.2 million people globally of which 1.2 million endure irreversible blindness [3].

The WHO strategy to eliminate all avoidable blindness by the year 2020 is called VISION 2020: Right to Sight. Blinding trachoma as a public health problem is specifically targeted as a priority condition in this initiative through an alliance of partners working with WHO called the Global Elimination of Trachoma as a Cause of Blindness by the Year 2020 (GET 2020) [4]. These collaborators work toward eliminating trachoma-related blindness through the implementation of an integrated intervention called the SAFE strategy (surgery, antibiotic, face washing, and environmental control); it is a multifaceted methodical initiative attempting to stop all levels of disease transmission and eliminate its reoccurrence [16]. The SAFE strategy includes the following: prevention of blindness by treating end-stage trachoma through surgery; reduction of the reservoir of infection by distributing antibiotics such as azithromycin; and removal of risk factors that encourage transmission of infection through facial cleanliness and environmental changes [17].

The “A”, “F,” and “E” components of the SAFE strategy focus on the reduction of disease transmission. Because active trachoma clusters in households or social groups, infection control should consist of community-based efforts to interrupt transmission as evidenced through the WHO-recommended mass antibiotic treatment of an entire district population annually for three years or more if active trachoma prevalence is above 10% [18]. Due to the inclusive nature of MDA programs, they are successful in reducing the prevalence of infection, slowing trachoma transmission and making it more manageable by WASH components to decrease the likelihood of reemergence as a public-health problem [19]. Before azithromycin, an ingestible antibiotic, for trachoma control, tetracycline eye ointment was used as the antibiotic component of the SAFE strategy [20]. Tetracycline compliance, and therefore effectiveness, was affected by its tedious application process, twice a day for six weeks [21]. Azithromycin treatments are easily administered by community control programs through height-based dosing and are considered by some to be the closest to the perfect antibacterial for MDA [22, 23]. 

In the following research, a systematic review was conducted to look a step beyond the impact of the A, F, and E components on the overall prevalence of trachoma and focus on the added value of WASH interventions, specifically the components “F” and “E” of the SAFE strategy, on the “A” component.

## 2. Methodology

### 2.1. Search Strategy and Initial Selection Criteria

A systematic search and review of articles was conducted using the Meta-analysis of Observational Studies in Epidemiology (MOOSE) as a guideline [24]. PubMed, Lista, EBSCO, and MEDLINE databases were searched using key words pairing water, sanitation, and hygiene education against trachoma and either preventive chemotherapy, mass chemotherapy, or antibiotic. No restrictions were put on study date, location, design, or language of publication. The final search was conducted on March 20, 2013.

The primary outcome measure for this review was prevalence of active trachoma measured as the number of participants with trachomatis inflammation follicular (TF) and/or trachomatis inflammation intense (TI) before and after intervention. Active trachoma was identified by the WHO trachoma grading scale and/or diagnostic swab testing for ocular Chlamydia infection [25]. Secondary outcomes included changes in knowledge, attitudes, and practice specific to the risk factors associated with prevention of trachoma through antibiotics, face washing, and environmental control.

Eligible study designs included peer-reviewed studies evaluating the impact of WASH interventions on the antibiotic component of the SAFE strategy. All articles included in the review were required to report trachoma prevalence data before and after program implementation. Any studies focused specifically on the outcomes of WASH interventions as an added value to MDA with azithromycin treatments were included. Due to limitations of monitoring and lack of compliance with the use of tetracycline, studies distributing tetracycline and not azithromycin to people with active trachoma were excluded. All impact studies, providing a complete assessment of the SAFE strategy and its impact on trachoma prevalence, rather than the added benefits of the interventions to one another were additionally excluded. Other excluded articles were those that focused on analyzing risk factors for trachoma and providing descriptions of trachoma and SAFE strategy through the literature-based review.

### 2.2. Data Collection and Analysis

Titles and abstracts found in the electronic databases were screened for relevancy by one author. An external source familiar with the subject matter was consulted when questions arose regarding a study's eligibility. Abstracts were examined from each search result and any abstract describing risk factors, survey results of MDA specifically or providing a general overview of trachoma was automatically discarded. Full copies of papers including the combined use of key words including SAFE, WASH, intervention, impact, added value, MDA, and azithromycin were obtained for review. As studies were identified for inclusion into the review, their listed references, via abstracts, were examined for potential relevant studies. One abstract was unavailable for review through the databases as well as interlibrary loan; this study was excluded based on the lack of relevancy of the title. All studies meeting the initial selection criteria by reporting trachoma prevalence data before and after program implementation and focusing specifically on the outcomes of WASH interventions as an added value to mass drug administration (MDA) of azithromycin treatments were included. Once all titles were assessed, the relevant articles' full texts were retrieved and relevant information was extracted onto a standardized form then pooled for summary estimates of the effectiveness of the interventions on the preventive chemotherapy programs specific to trachoma. The data fields included on the extraction form are presented as follows:

 Author
 Title
 Journal
 Date
 Study type
 Year of Baseline Survey
 Sample size (*n*) at baseline
 Trachoma grading: WHO, diagnostic, or both
 Specific Intervention
 Specific antibiotic treatment
 Population group
 Age range
 Number of communities
 Country
 Region
 District
 Year of followup
 Sample size (*n*) at followup
 Baseline prevalence
 Postintervention prevalence
 Effect measure
 Estimate
 95% confidence interval
 
*P* value.

## 3. Results

### 3.1. Results of the Search

**Figure 1:**
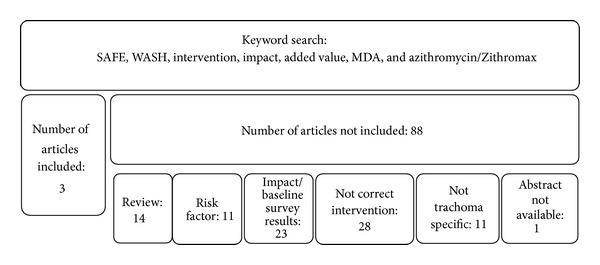
Breakdown of nonduplicated searches by reason for exclusion. Not correct intervention: either no WASH or MDA components were mentioned or tetracycline was used rather than azithromycin.

The electronic searches yielded 156 abstracts; a total of 91 were nonrepeating. All nonrepeating searches were screened by one author on two different occasions. After the screening, the full texts of 10 relevant articles were retrieved. These articles were assessed by one author; any questions arising from the assessment of these articles were answered by an external source specialized in NTDs (Figure 1). 

From the 10 articles retrieved, one study of Edwards 2006 did not include antibiotic treatment due to limitations in distribution at the time of the study and therefore was excluded from the review [7]. However, this study was followed up again two years later by Cumberland 2008 and with the correct inclusion criteria those results were included in the review [13]. Two other articles by Astle 2006 and Khandekar 2005 were not included because the antibiotic distribution was not in the capacity of an MDA to the entire community and was only distributed to certain individuals during trachoma prevalence screening [6, 8, 10]. Four more articles evaluating the comprehensive SAFE strategy Ngondi 2006, 2008 and 2010 as well as Roba 2010 were excluded because the assessment did not specifically look at the added value of the SAFE components to each other [8–12, 14]. The remaining three studies were considered and accepted for inclusion in the review (Cumberland 2008, Khandekar 2006, and Lansingh 2010) [13–15]. A full list of reviewed excluded studies is included in Table 1.

### 3.2. Setting and Participants

#### 3.2.1. Including Type of Study, Unit of Randomization, Place, and Number

The three eligible studies were diverse in population, region, study design, and intervention. All were intervention studies, with a specific intention to study the added value of the F and E components of the SAFE strategy.

### 3.3. Types of Participants

Participants in these trials were residents in trachoma endemic communities, in Ethiopia, Vietnam, and Australia. No age restriction was placed on the studies; however because active trachoma is usually diagnosed in children under 15 years of age, the studies self-limited themselves to children below the age of 15.

*Ethiopia.* The Cumberland et al. 2008 study was a randomized controlled trial with staged interventions, beginning in Guarage, Oromia, and South Welo districts of Ethiopia between 2002 and 2003 [13]. A total of 1,722 participants, 3–9 years of age, in 37 communities were randomized into three intervention groups and one control group. Five communities were used in the control group and 32 received combined intervention activities to serve as the intervention group. Because 33 children with TI did not have follicles, the final analysis was based on a total of 1,689 children.

*Vietnam.* The Khandekar et al. 2006 study was a community-based health intervention study, whereby an intervention community was compared to a nonintervention community, conducted in two communities, My Thon, the intervention village, and Xom Ngoia, the nonintervention village, in the Gia Binh district of Vietnam [14]. In March of 2002, children aged less than 15 years old in each community were evaluated for inclusion in the study. Participants were chosen by random selection. The total number of children assessed in the analysis of the study was 911.

*Australia.* The Lansingh et al. 2010 study a prospective case study, was community-based study lasting 12 months from 2000 to 2001, including two hyperendemic Aboriginal Australian communities [15]. Both communities assessed children <15 years of age and were matched at baseline with regards to population age, gender, trachoma disease markers, and desert climate and environment. Community 1, the intervention community, had 86 participants and community 2, the nonintervention community, had 91. After 3 months of intervention the total number of children under 15 years of age reassessed was 107, after 6 months the total increased to 111 and after 12 months the total was 165.

### 3.4. Interventions

#### 3.4.1. Included Types, Setting, and Duration

In the Cumberland 2008 study populations in the control villages only received radio broadcasts as a hygiene education intervention. In addition to radio broadcast both intervention groups received antibiotics as well as health information in the form of information education and communication materials (IEC). One group additionally received drama and video. Though all intervention communities received MDA, approximately half of the intervention communities received azithromycin 2–5 months prior to the survey and most had received more than one dose. The interventions were ongoing for three years.

In the Khandekar 2006 study the nonintervention community implemented the “S” and “A” components of the SAFE. In the intervention village, the full SAFE strategy was implemented. The additional components specific to “F” and E” included the provision of clean water and sanitary latrines as well as health behavior education. Some specific interventions included but were not limited to performances, billboards of trachoma-control messaging, latrines, dug wells, and water filters. The postintervention followup occurred in 2005, three years after the interventions began.

In Lansingh 2010, health and facial cleanliness promotion and azithromycin were distributed to all members of both communities. The intervention community received an additional E component intervention through housing and environmental improvements. Some of the specific improvement categories included biweekly trash collection, upgrades in sewage and water lines, and installation of rainwater tanks.

### 3.5. Outcomes Measures

In the Cumberland 2008 study outcome measures recorded in the study included prevalence of active trachoma, TF only in one or both eyes, at baseline and then again after intervention. The clinical diagnosis of active trachoma was graded using the WHO-simplified grading system. Additionally, eye swabs of all children were taken from the right eye to detect the presence of polymerase chain reaction (PCR) in ocular secretions *C. trachomatis* deoxyribonucleic acid (DNA). 

Secondary outcomes examined knowledge and behavior related to trachoma control. These were measured through observations and questionnaires. Observations were made by field workers of household facilities, cleanliness, and presence of flies in and around the houses. Questionnaires were delivered by a locally recruited interviewer to an adult care-giver in the home. Questions included demographic details of both respondents and household; they were specific to care of livestock, knowledge of health-related issues, and practical arrangements for sanitation which were supported by observations of field workers of beaten paths to the latrines and human faeces in the pit.

In the Khandekar 2006 study both eyes of all family members were examined using the WHO trachoma grading scale; if trachoma was reported in either eye, the person was considered to be suffering from active trachoma, TF/TI.

Secondary outcomes were assessed through close-ended questionnaires measuring the knowledge, attitude, and practices regarding trachoma among mothers in both villages. Questions related to attitude and practice had five grades, from fully agree to entirely disagree, and questions related to knowledge included common symptoms of trachoma, blinding complications of trachoma, methods of methods of prevention, water sources for drinking and face washing, and the advantages of a sanitary latrine. Evaluation of water and sanitation status was done by a water engineer who quantitatively and qualitatively recorded the status of the water, latrine, and other sanitation facilities in and around the house.

In the Lansingh 2010 study baseline measures of trachoma prevalence, facial cleanliness, and nasal discharge were measured. Assessments of trachoma and facial cleanliness were made at 3, 6, and 12 months before intervention. Clinical assessment of trachoma was conducted by a single examiner using the WHO-simplified grading system. Active trachoma was defined as the presence of TF or TI. 

Secondary measures were taken by Aboriginal Health workers (AHW) trained to assist with examinations, promote health education when participants visited clinics for any reason, and conduct the health education campaign at the home level. An active health education campaign with emphasis on facial cleanliness was instituted in schools, primarily facilitated by teachers and personnel from the Nganampa Health Council. 

Absolute facial cleanliness was defined as the absence of ocular and nasal discharge. Partial facial cleanliness was defined as having either ocular or nasal discharge, but not both. A “dirty face” had both ocular and nasal discharge; the quality of discharge was also recorded as clear, abundant, or mucopurulent. Health Living Practices (HLP) were measured by survey at baseline and approximately 8 months after intervention, to be considered passing, eight of the nine HLP must be met.

### 3.6. Effects of Intervention

Due to the statistical, methodological, and clinical heterogeneity of studies conducting a meta-analysis was not considered appropriate and a narrative summary of the results is presented.

### 3.7. Primary Outcome: Prevalence of Active Trachoma

In Cumberland 2008 study, the overall prevalence (standard deviation: SD) of active trachoma in the 37 communities were 71.4% (17.6) in 2002 and 35.6% (17.4) in 2005, illustrating around a 50% reduction in prevalence. The cluster summarized prevalences (SD) of active trachoma estimated in the control communities were 60.7% (12.1) in 2002, 55.1% (12.5) in 2003, and 54.5% (20.3) in 2005. The reduction in the intervention community was found statistically significant but the reduction in the control communities, only receiving radio as an intervention, was not statistically significant (*P* = 0.692). The odds of trachoma in the intervention communities, after intervention, are summarized in Tables 2 and 6.

In communities receiving azithromycin and video educational interventions, a reduction in the odds of active trachoma did not occur. However, after adjustment for age, results suggested an almost 70% reduction in odds of active trachoma in children living in communities that received antibiotic treatment and IEC health education materials and those in communities that additionally received the video health messages. 

In Khandekar 2006 study, the village with the added value of the F and E components experienced a decrease in the prevalence of trachoma, in children <15 years, from 13.8% at baseline to 2.3% after intervention, a prevalence difference of 11.53%. Prevalence of active trachoma dropped as well in children <15 years from the nonintervention community (SA only) from 10.2% to 5.54%, an absolute difference of 4.7%. The additional absolute decline of trachoma infection due to F and E in children <15 years of age was 6.8%.

The decline of active trachoma in the nonintervention village was considered to be due to the impact of the S and A components. When the impact of the F and E strategy under the circumstances of this study was controlled for, a decline of 59.2% in active trachoma was attributed to the additional activities of the F and E components of the SAFE strategy in the intervention community. (6.83/11.53 ∗ 100 = 59.2%).

In Lansingh 2010 study a reduction in prevalence of trachoma was observed three months after antibiotic administration. At that time, the active trachoma prevalence reduced from 47.7% to 21.2% in the intervention community and from 49.6% to 24.2% in the nonintervention community. By 12 months it rose to 30% in the nonintervention community but remained stable in the intervention community. The reduction in trachoma prevalence between preintervention and 12 months after intervention was significant for both communities:

 
*X* ^2^ = 9.1 Intervention Community (A, F, E) *P* = .003.
 
*X* ^2^ = 8.1 Nonintervention Community (A, F) 2*P* = .005.

However, there was no significant difference in the trachoma prevalence between the two communities at any examination.

Table 3 illustrates a synopsis of results in change of trachoma prevalence.

### 3.8. Secondary Outcome 

#### 3.8.1. Changes in Knowledge, Attitude, and Practice

In the Cumberland 2008 study, there was no evidence of a difference in awareness of trachoma and trichiasis across intervention arms; however, the overall community-summarized awareness was greater than 80%. Since the initial baseline survey in 2002 and the follow-up survey in 2003, householders reported knowing at least one trachoma prevention method as compared to not knowing any in communities receiving printed and video health education. 

In the Khandekar 2006 study, the knowledge, attitude and practices among the mothers of both villages at baseline and at the end of two years were compared. An improvement in knowledge of the subcomponents of trachoma prevention, including knowledge of trachoma, among the mothers in the intervention village was greater than among the mothers in the nonintervention village. However, the attitude and practices of trachoma control continued to be at the same level among the mothers of both villages.

The Lansingh 2010 study did not measure knowledge, attitude and practices as a secondary outcome for trachoma knowledge; instead, the study looked specifically at facial cleanliness in regards to a measure of the added benefit of the “E” intervention. Postintervention followup at 12 months showed a greater percentage of clean faces and less prevalence of nasal discharge in the non-“E” intervention community. 

## 4. Discussion and Conclusion

### 4.1. Trachoma Prevalence

Poverty creates a major barrier to gaining access to clean water, perpetuating the link between trachoma, lack of water, sanitation, hygiene education, and economic development [26]. According to the WHO, “the availability of clean water is a prerequisite to the sustainable growth and development of communities around the world” [27]. The conditions of poverty and inequality perpetuating the prevalence of trachoma and unsafe water and sanitation continue claiming lives, destroying livelihoods, and compromising prospects for economic growth [26]. Economic stability affects poor living conditions increasing the need for environmental interventions in trachoma control [22]. If all associated factors important to transmission of trachoma are not addressed, then a sustainable program cannot be created and the disease is likely to recur [28]. As a result, sustainable control and elimination strategies for trachoma are especially reliant on the development of the overall infrastructure of a nation through the integration of collaborative efforts between the health and WASH sectors [29]. This review tried to recognize the added value of WASH factors to the “A” component of the SAFE strategy in order to advocate for collaboration between sectors for trachoma control ultimately elevating the continued cycle of poverty propagated by trachoma [20].

The articles included in the review observed a decrease in prevalence of trachoma over the entire length of study. Although the magnitude of effect varied, the decline in trachoma was apparent in all villages, both control and intervention for all three studies reviewed in the control communities. Where no additional interventions took place, the decrease was due to the natural improvements in socioeconomic status [30]. Even without intervention, change in village demographics and employment brought additional income resulting in access to water and sanitation facilities as well as increased access to mass media through additional purchasing of radio and TV.

### 4.2. Primary Outcome

In the Cumberland 2008 and Khandekar 2006 study, the decrease in trachoma prevalence was significant not only between the pre- and postinterventions but between the intervention and nonintervention communities. This suggests that adding water, sanitation, and hygiene education interventions to the ongoing mass antibiotic distribution had a significant impact on reducing the prevalence of trachoma. In contrast, however, the Lansingh 2010 study found a significant decline between pre- and postintervention but not between villages. The study was unable to determine if the “E” environmental component added significant value to the SAFE strategy between the two villages studied. There are many reasons for the variability in results between these studies including quality of study design and coverage rates of antibiotic. 

In Cumberland 2008 study, the decline in prevalence was attributed to treatment of azithromycin; however, the other components studied as well reported added value to the decline of trachoma prevalence, around a 50% decrease from baseline to postintervention followup. The study suggested that health education and community based programs appeared to have additional positive results on the “A” component of SAFE, but percentage prevalence decreased because of the AFE components were not separately calculated. The Khandekar 2006 study did calculate the added benefit and found that providing the “F” and “E” components in addition to the “S” and “A” contributed to a nearly 60% decline in active trachoma prevalence. These components were additionally responsible for the decline of more than one-third of the active trachoma prevalence in children, confirming the association of water and sanitation improvement with the decline of active trachoma.

In the Lansingh 2010 case study, the “A” and “F” components of the SAFE strategy interventions significantly reduced the prevalence of trachoma in both communities. Though the communities achieved significant reductions in trachoma prevalence between preintervention and 12-month postintervention assessments, there was no evidence that the specific environmental changes implemented in the intervention community had any additional impact to the improvement of facial cleanliness and antibiotic distribution. The reason for the lack of significant change was due to the small sample size, low antibiotic coverage, and problems with the overall study design. 

### 4.3. Secondary Outcome

#### 4.3.1. Knowledge, Practice, and Attitude (KPA)

Changing deep-rooted cultural behaviors is difficult; however, if effective, it creates a collective and sustainable awareness [31]. In Cumberland 2008 study, the percentage of householders surveyed who knew about trachoma was 45%; of those only 20% knew about trachoma risk factors and treatment options. Previously, the emphasis for prevention and treatment methods for trachoma focused on traditional beliefs and practices. It was reported that in 2003 and 2005, a shift occurred away from traditional beliefs to a better understanding of trachoma and the SAFE strategy as a preventative method and cure.

In the Khandekar 2006 study, a notable improvement in the KAP of mothers after two years was demonstrated. The reason for the improvement was due to the leveraging of organizations within the village in addition to passive advocacy through mass media. The model included using women's associations and involving school teachers to educate the community. Lansingh 2010, did not measure KAP as an outcome of the added benefit of the “E” component.

#### 4.3.2. Facial Cleanliness and Nasal Discharge

In the Lansingh 2010 study, contrary to the assumed outcome, the nonintervention community which received only the “A” and “F” components of SAFE performed better than intervention community, which had the additional “E” component, in regards to facial cleanliness and nasal discharge. These secondary outcomes, however, were not easily correlated to the small difference in prevalence of trachoma.

### 4.4. Quality of the Evidence

Establishing a reliable study design is crucial in providing quality evidence; yet it is always a challenge. To minimize risk of bias, a study has to establish appropriate sample size, randomize participants, control for confounders, and select controls to match cases [9]. In this review, the study with the most comprehensive description of these elements was by Khandekar 2006. In the Cumberland 2008 and Lansingh 2010 studies it was unclear if risk of selection/sampling and confounding bias was accounted for or if it was simply omitted in the reporting stage. In regards to this review, the analysis was conducted with information provided only by the respective articles.

### 4.5. Selection Bias

Of the three studies reviewed, one study, the Lansingh 2010 study, did not mention randomizing participants. The Cumberland 2008 study stated that the participants and communities were randomly chosen. Delays with distribution prevented the study to stick to the original randomization schedule and local NGO's were responsible for distribution of interventions on their own schedule. The details of the original design were reported in the 2003 study published in 2006. The Khandekar 2006 study also stated random selection. The researchers also tried to minimize systematic error by having an appropriately sized population of all ages. In contrast, the Lansingh 2010 study did mention that the effect size (which at the time had not been tested for the E component) was beyond the detectable level since the number of children surveyed in both communities was relatively low. 

### 4.6. Comparability

In the Cumberland 2008 study, the communities used in the study were randomly selected from different zones within the trachoma control program areas. There was, however, no additional information offered about the comparability of these villages. In the Khandekar 2006 and Lansingh 2010 studies the studied villages were chosen specifically because of comparable characteristics. In the Khandekar 2008 study the villages had similar population profiles and sanitation status; only the water sources differed. only the water sources differed. In order to prevent cross-contamination between groups, study villages were in separate communities with five other villages between them and markets and educational centers were also different. In the Lansingh 2010 study, the two communities were matched in general at baseline with regard to population age, gender, trachoma disease markers, desert climate and environment, although mobility was much higher in the intervention community.

### 4.7. Compliance

#### 4.7.1. Azithromycin Coverage

During mass drug administration (MDA) of azithromycin 100% of the eligible population is targeted for treatment through preventive chemotherapy. Coverage results above 90% are considered reliable in achieving intervention outcomes and reducing the chance for recurrence of trachoma [20]. In the Cumberland 2008 study, 92% coverage of azithromycin distribution was reported. The Lansingh 2010 study, only achieved an antibiotic coverage of 73% and 55% in communities 1 and 2, respectively, and the Khandekar 2006 study did not specify MDA coverage rates.

#### 4.7.2. Compliance with Water and Sanitation

Compliance of the “F” and “E” components was measured by frequency of face washing, latrine use, and other sanitation interventions.

In the Cumberland 2008 study, householders in intervention villages reported that additional family members used pit latrines for defecation, and 60–70% of all householders in each arms of the study reported good rubbish disposal practices. In intervention communities there was a 15% decrease of community-reported prevalence of animal faeces in the immediate proximity to the house.

In the Khandekar 2006 study access to a sanitary latrine and use by all members of the family (including children) improved in both villages in two years but it was better in the intervention village than in nonintervention village. Additionally, fly density was significantly lower in the intervention village than in the nonintervention village at the end of two years:

 
*X* ^2^ = 270, df = 2 *P* = 0.00001.

Water facilities improved in both villages after two years of intervention. However, in the nonintervention village the quality of water and cleanliness around the water source remained unsatisfactory in about one-third of the houses.

In the Lansingh 2010 study, baseline prevalences of an absolutely clean face in the intervention community were 30% and 23% in the nonintervention community while those of a dirty face were 64% and 58% respectively (difference not significant). After the intervention, in children less than 10 years, no nasal discharge was observed on 30% of faces in the intervention community compared to 12% in the nonintervention community:

 
*X* ^2^ = 5.7*P* = 0.02.

The intervention community showed positive postintervention Nganampa Health Council (NHC) survey results with substantial gains in washing clothes/bedding (+52% change) and removing waste safely (+14% change) following the intervention; however, the majority of houses did not reach a change in outcomes in most categories.

### 4.8. Confounding

In order to minimize misrepresentation of the overall effects of the added value of the WASH components, the studies controlled for confounding variables known to affect the prevalence of trachoma. In the Khandekar 2006 study, the following variables were collected and controlled for age, gender, educational level, occupation, level of poverty, and access to mass media. While active trachoma rates were projected for the study population, the rates by age and gender were adjusted to compensate for the differential representation of subgroups. The effects of these confounders were found to be minimal in this study. In both Khandekar 2006 and Lansingh 2010 studies, climate was considered a possible confounder. It was observed that trachoma prevalence was higher during certain seasons. For this reason both studies planned prevalence assessments at similar times and in similar conditions. 

Though the Lansingh 2010 study accounted for climate, other potential confounders were not controlled for. The levels of dust and flies were not assessed in the nonintervention community, and the houses were not surveyed before or after the intervention. By not controlling for these confounders, there was no valid information of the number of houses that might have passed the various HLP categories in the nonintervention community, and whether the A/F interventions there made any difference. Cumberland made no mention of controlling for confounders in the 2008 study design.

### 4.9. Masking

The Khandekar 2006 study was the only study to specifically mention masking as a way to minimize observer bias. Though the study was unable to mask the field staff that assessed the status of the water and sanitation, the results in the houses of the intervention village and the nonintervention village were coded and given to an external evaluator. 

### 4.10. Applicability

Factors affecting generalizability of methods were different for each study and a summary of methodological elements is presented in Table 4. The Cumberland 2008 study found additional value to health education yet acknowledged that educational campaigns are time consuming and produce variable results. Additionally, the community mass media intervention used does not present an effective method of ensuring sustainable prevention in hyperendemic rural populations which may have limited access to mass media including radios. Control for sampling bias was considered in the study design, even though it was reported in a previous article. The methods used in the Khandekar 2006 study, including leveraging local organizations with skills of social marketing and community commitment, could be promoted in other rural areas of developing countries as a form of trachoma control. The Lansingh 2010 study, however, lacked complete applicability of evidence due to the previously mentioned absence of a reliable and valid study design (Table 5). 

### 4.11. Authors' Conclusions

#### 4.11.1. Implication for Practice

In order to eliminate blinding trachoma as a public health problem, recurrence of the active form of the disease must be interrupted before repeated scarring leads to trichiasis. The antibiotic component of the SAFE strategy offers an immediate treatment option; however it is not considered sustainable [19]. Combining the “F” and “E” components strengthens the prevention and control of trachoma; yet little research has been done on the actual amount of added value the individual “A,” “F,” and “E” components have to one another. After thorough review of the literature, all articles showed the “F” and “E” components provided significant value to the overall decrease of prevalence of active trachoma. However, due to study limitations and an insufficient number of articles on the subject, it is not possible to determine the added benefit of the “F” and “E” components to the mass distribution of azithromycin as the “A” component. Additional research in identifying the added value of WASH on decreasing trachoma infection would facilitate advocacy efforts and collaboration between trachoma programs with WASH groups to reach program goals. Establishing the added value for WASH components would encourage integration between appropriate partners in order to pool resources and use coordinated sites and indicators allowing both WASH and trachoma programs to amplify the impact of their interventions.

## Figures and Tables

**Table 1:** Excluded studies and reason for exclusion.

Author and year	Title	Reason for exclusion
Astle et al., 2006 [6]	Trachoma control in Southern Zambia—an international team project employing the SAFE strategy	Spot treatment
Edwards et al., 2006 [7]	Impact of health education on active trachoma in hyperendemic rural communities in Ethiopia	No MDA
Khandekar et al., 2005 [8]	Active trachoma, face washing (F), and environmental improvement (E) in a high-risk population in Oman	Spot treatment
Ngondi et al., 2006 [9]	Effect of 3 years of SAFE (surgery, antibiotics, facial cleanliness, and environmental change) strategy for trachoma control in southern Sudan: a cross-sectional study	Impact Survey
Ngondi et al., 2008 [10]	Associations between active trachoma and community intervention with antibiotics, facial cleanliness, and Environmental improvement (A, F, E).	Assessed risk factors
Ngondi et al., 2010 [11]	Estimation of effects of community intervention with antibiotics, facial cleanliness, and environmental improvement (A, F, E) in five districts of Ethiopia hyperendemic for trachoma	Impact survey
Roba et al., 2011 [12]	Effects of intervention with the SAFE strategy on trachoma across Ethiopia	Impact survey

**Table 2:** Summary of odds of trachoma after intervention in Cumberland 2008.

Intervention-only communities (Does not include control village)	Age	Odds ratio	95% CI	Significance
Antibiotic treatment IEC health education video health	All	0.93	0.42–2.07	*P* = 0.086
Antibiotic treatment IEC health education	Children ages: 3–9	0.35	0.13–.089	*P* = 0.027
Antibiotic treatment IEC health education video health	Children ages: 3–9	0.31	0.11–.089	*P* = 0.029

**Table 3:** Reported reduction of trachoma prevalence.

Reference	Length of study	Baseline trachoma prevalence		After intervention	Percent reduction	Added value
Cumberland et al., 2008 [13]	3 years	Control	60.7%	54.5%	10.0%	59.2%
		Overall	71.4%	35.6%	50.0%	
Khandekar et al., 2006 [14]	3 years	Control	10.2%	5.54%	46.1%	
		Intervention	13.8%	02.3%	83.0%	
Lansingh et al., 2010 [15]	1 year	Control	49.6%	30.0%		
		Intervention	47.7%	21.2%	

**Table 4:** Reported increase in primary trachoma-related KAP.

Reference	Secondary outcome measure	Baseline KAP		After intervention KAP		Percent Increase
Cumberland et al., 2008 [13]	Knowledge of trachoma	Overall	45.0%	Control	82.9%	48.0%
				Intervention 1	85.6%	47.0%
				Intervention 2	96.0%	54.0%
Khandekar et al., 2006 [14]	Knowledge of trachoma	Control	38.0%		78.0%	51.0%
		Intervention	45.4%		99.3%	54.0%
Lansingh et al., 2010 [15]	Facial cleanliness (absolutely clean 1-9 years)	Control	23.5%		57.0%	59.0%
		Intervention	16.5%		85.0%	81.0%

**Table 5:** Methodological evaluation of the quality of included studies.

Quality of evidence	Cumberland et al., 2008 [13]	Khandekar et al., 2006 [14]	Lansingh et al., 2010 [15]
Selection bias			
Random sampling	Yes	Yes	Yes
Sample size	N/A	Yes	No
Comparability	No	Yes	Yes
Compliance			
“A” coverage (over 80%)	Yes	N/A	No
“F” and “E”	Yes	Yes	Yes
Confounders			
Descriptive variables	No	Yes	No
Climate	No	Yes	Yes
Masking	No	Yes	No
Applicability	Yes	Yes	No

**Table 6:** Summary of included studies and health measures.

Paper	Intervention	Country and population	Study quality	Health outcome	Age group	Measure	Statistical test
Cumberland et al., 2008 [13]	Control: only radio MDA and radio MDA, radio and information education and communication materials (IEC) MDA, IEC, and community video and drama	Ethiopia 1689	Good	Trachoma	3–9 years	Change in prevalence	OR 0.35 radio, MDA, IEC CI 0.13–0.89 *P* value: 0.027 OR 0.31 radio, MDA, IEC, and video CI 0.022–0.89 *P* value: 0.029

Khandekar et al., 2006 [14]	Water supply Sanitation supply	Vietnam 911	Great	Trachoma	<15	Change in prevalence	

Lansingh et al., 2010 [15]	Environmental enhancements Water supply	Australia 111	Poor	Trachoma	<15	Change in prevalence	*X*² = 9.1 community 1 *P* value: 0.003 *X*² = 8.1 community 2 *P* Value: 0.005 OR 0.99 CI 0.93–1.05
